# Predictive value of non-fasting remnant cholesterol for short-term outcome of diabetics with new-onset stable coronary artery disease

**DOI:** 10.1186/s12944-017-0410-0

**Published:** 2017-01-13

**Authors:** Li-Feng Hong, Xiao-Ni Yan, Zhen-Hua Lu, Ying Fan, Fei Ye, Qiong Wu, Song-Hui Luo, Bo Yang, Jian-Jun Li

**Affiliations:** 1 Department of Cardiology, the Fifth Hospital of Wuhan & Cardiovascular Institute of Jianghan University, Wuhan, 430050 China; 2 Department of Cardiology, Renmin Hospital of Wuhan University, Wuhan, China; 3 Division of Dyslipidemia, State Key Laboratory of Cardiovascular Disease, Fu Wai Hospital, National Center for Cardiovascular Diseases, Chinese Academy of Medical Sciences, Peking Union Medical College, Beijing, China

**Keywords:** Remnant cholesterol, Low density lipoprotein cholesterol, Diabetic mellitus, Coronary artery disease, Outcome

## Abstract

**Background:**

The relationship between non-fasting remnant cholesterol and cardiovascular outcome in the era of potent statin therapy remained to be elucidated.

**Methods:**

A cohort study of three hundred and twenty eight diabetics diagnosed with new-onset stable coronary artery disease (CAD) by coronary angiography were enrolled. All cases were followed up for an average duration of twelve months. The association between baseline remnant cholesterol levels and major cardiovascular outcomes were evaluated using the receivers operating characteristic (ROC) curves and Cox proportional hazards regression analysis.

**Results:**

During a period of 12-month’s follow-up, 14.3% patients (47/328) underwent pre-specified adverse outcomes. The remnant cholesterol associated with high sensitivity C-reactive protein, neutrophil count and fibrinogen (*R* ^2^ = 0.20, 0.12 and 0.14; *P* = 0.000, 0.036 and 0.010 respectively). Area under the ROC curves (AUC) indicated discriminatory power of the remnant cholesterol to predict the adverse outcomes for this population (AUC = 0.64, *P* < 0.005). Kaplan-Meier curve suggested that the lower levels of remnant cholesterol showed relatively lower cardiac events for diabetic patients with stable CAD (Log rank *X* ^2^ = 8.94, *P* = 0.04). However, according to multivariate Cox proportional hazards regression, apart from hemoglobin A1C (Hazard ratio [H.R.] =1.38, 95% CI: 1.14–1.66, *P* = 0.001) and Gensini scores (H.R. = 1.00, 95% CI: 1.00–1.02; *P* = 0.035), remnant cholesterol did not qualify as an independent predictor of adverse prognosis in these settings (H.R. = 1.05, 95% CI: 0.46–2.37, *P* = 0.909).

**Conclusions:**

Non-fasting remnant cholesterol was associated with inflammatory biomarkers and high incidence of revascularization, but not qualified as an independent predictor for short-term prognosis of diabetics with new-onset stable coronary artery disease.

## Background

Remnant cholesterol is the cholesterol content of triglyceride-rich lipoproteins, composed with very low density lipoproteins and intermediate density lipoproteins in the fasting state and of these 2 lipoproteins together with chylomicron remnants in the non-fasting state [1, 2]. Concerning to our previous investigations, substantial evidence from clinical, general population and genetic studies have led to a consensus that the cholesterol transported by remnant lipoproteins or so called remnant cholesterol is powerful contributor for both coronary artery disease (CAD) and stroke risk [3–6]. Moreover, it is gradually clarified that irrespective of the fasting status, defined, and measured or calculated in different ways, remnant cholesterol is causally correlated to the risk of CAD and low-grade inflammation independent of HDL-C levels [7, 8].

Mechanistic studies have identified remnant cholesterol accumulating and infiltrating the endothelial barrier, spuring inflammatory reaction and atherogenic process in the arterial wall like LDL-C, and observational studies have also confirmed that elevated remnant cholesterol was correlated to risk of CAD [8]. By the virture of their larger size, remnant cholesterol carries 5 to 20 times as much cholesterol pe particle as LDL-C [9]. Importantly, unlike native LDL-C, remnant cholesterol can up-regulate the expression of scavenger receptors and thus promoting foam cell formation [10]. Thus, genetic studies of variants associated with elevated remnant cholesterol levels show that an increment of 1 mmol/L in levels of non-fasting remnant cholesterol associates with a 2.8-fold increased risk of CAD [8]. Although triglyceride per se are not qualified as a direct culprits in the atherogenic process, several prospective studies have suggested the robust predictive value of triglyceride both for mobility and mortality [11–16]. It is well-known non-fasting remnant cholesterol and triglyceride are highly correlated (*R* ^2^ = 0.96) [7]. Remnant cholesterol is an important component of non-HDL-C and non-LDL-C. Prior population studies have long been suggested that non-HDL-C is a more robust risk marker for cardiovascular disease than LDL-C alone [17, 18]. Furthermore, as a result of the high prevalence of comorbidities and conventional risk factors among patients with type 2 diabetes, most patients belong to the highest CAD risk category, and have substantial residual risk due to remnant cholesterol even when levels LDL-C persist in recommended target [19]. Thus, we wonder whether non-fasting remnant cholesterol is a useful indicator for cardiovascular prognosis for type 2 diabetics with new-onset stable CAD.

## Methods

### Study populations

From June 2011 through March 2012, a total of 328 type two diabetic patients with chronic stable angina pectoris (typical exertional chest pain exhibiting the same pattern for ≥3 months) were selected from among 1400 consecutive patients who underwent diagnostic coronary angiography (CAG). All enrolled patients were confirmed to have stenosis of not less than 50% in at least one main coronary vessel at our center. Patients with a history of revascularization or without obstructive coronary artery lesions and with type 1 diabetes mellitus (DM), acute coronary syndrome (ACS), significant hematologic disorders (white blood cell count ≤ 3.5*10^9^/L or ≥ 20*10^9^/L), infectious or inflammatory disease, various tumor, severe liver and/or renal insufficiency were excluded. All subjects enrolled were underwent detailed clinical, laboratory, and angiographic examination for assessment of the cardiac status and were asked for their present and past history about traditional risk factors of CAD such as smoking habits, hypertension, hyperlipidemia, obesity, diabetes mellitus, previous stroke, peripheral vascular disease, family history of CAD and non-cardiovascular diseases.

Hypertension was defined as repeated blood pressure measurements ≥140/90 mmHg and was assumed to be present in patients taking anti-hypertensive drugs. Diabetes mellitus was diagnosed in patients with fasting serum glucose level of ≥6.99 mmol/L in multiple determinations or under active treatment with insulin or oral hypoglycemic agents. The differentia of type 1 and type 2 DM was carried out by multiply elements such as the age of onset, history of ketosis, concentration of insulin, curves of insulin release, and clinical manifestation. Hyperlipidemia was considered to be present in patients with fasting total cholesterol (TC) ≥ 5.2 mmol/L or TG ≥ 1.7 mmol/L.

The stable angina pectoris conformed to Canadian Cardiovascular Society of grade I ~ III and excluded from ACS. The indications for CAG are in accordance with the ACC/AHA guidelines for CAG [20]. CAD was defined as the presence of significant obstructive stenosis, at least 50% of the vessel lumen diameters, in any of the main coronary arteries by at least two independent senior interventional cardiologists based on quantity coronary angiography (QCA). The severity of CAD was evaluated with revised Gensini score system [21]. Stent implantation, periprocedural medical treatment and care were performed according to standard criteria when there were indicative of revascularization. Post-interventional antiplatelet therapy consisted of clopidogrel and aspirin with formal dosage. Drug eluting stents were majorly implantation. The left ventricular ejection fraction was evaluated by echocardiographer using the area-length methods with modified Simpson’s rule.

### Measurements of biomarkers

Venous blood samples were obtained from each patient at baseline upon admission. Plasma TC and TG were measured by enzymatic methods and HDL-C by a direct method (Roche Diagnostics, Basel, Switzerland). Low-density lipoprotein cholesterol (LDL-C) was obtained by Friedewald’s formula (if plasma triglycerides < 4.0 mmol/l), and otherwise measured directly using ultracentrifugation. Non-fasting remnant cholesterol was calculated as nonfasting TC minus HDL-C minus LDL-C. Apoprotein A-1 (ApoA-1) and ApoB were measured by an immunoturbidimetric method (Tina-quant, Roche Diagnostics) calibrated against the World Health Organization/International Federation of Clinical Chemistry reference standard SP3–07. The levels of high-sensitivity-CRP were determined using immunoturbidimetry (Beckmann Assay 360, Bera, Calif., USA) according to our previously reported. The levels of hemoglobin A1C (HbA1C) were measured using the Tosoh G7 Automate HPLC Analyzer (TOSOH Bioscience, Japan). All other included biomarkers were analyzed by standard biochemical tests, as previously reported [22].

### Follow up and study endpoints

The follow-up protocol after discharge of enrolled patients consisted of a phone or clinic interview. Patients were followed up for an average duration of 12 months. During the period, no individual was lost to follow-up. The pre-specified clinical end points were defined as cardiac causative death or cardiac death, nonfatal myocardial infarction (MI), revascularization, and re-hospitalization due to attack of ACS.

### Statistical analysis

All Statistical studies were performed with the SPSS program (version 19.0, SPSS, Chicago, Illinois, USA).

The Kolmogorov-Smirnov test was used to test the distribution pattern. Quantitative variables were expressed as mean ± standard deviation (SD), and qualitative variables were expressed as numbers and percentages. According to the present or absent of pre-specified cardiovascular outcome, the enrolled population was categorized into two groups (outcome present group, *n* = 47; outcome absent group, *n* = 281). Continuous variables were analyzed using either Student’s *t*-test or the ANOVA methods (normal distribution) or the Mann-Whiteny *U*-test or Kruskal-Wallis test (abnormal distribution). Categorical variables were compared using chi-squared statistic tests. The relationship among the remnant cholesterol and other lipid parameters with inflammatory biomarkers, HbA1C and Gensini scores were assessed using Spearman’s correlation analysis. Kaplan-Meier survival curves and receiver operating characteristic (ROC) curve were all constructed aimed to demonstrate the discriminatory power of the remnant cholesterol for pre-specified cardiovascular outcome. In order to demonstrate whether the remnant cholesterol could independently provide prognostic information for diabetic patients with new-onset stable CAD, univariate and multivariate Cox proportional hazards regression analysis were performed. A *p* value of less than 0.05 was considered as statistically significant.

## Results

### Baseline characteristics of the study population

The cohort consisted of 240 men (73.3%) and 88 (26.7%) women aged 34–82 years (mean age 59.4 years). All enrolled subjects were diagnosed with type 2 DM and new-onset stable angina pectoris and referred to CAG. All patients were received an average of 12-month’s follow-up (ranged from 20 to 448 days).

The baseline demographic, clinical characteristics and angiographic findings of the enrolled subjects by the present or absent of pre-specified cardiovascular outcomes were summarized in Table 1. Noticeably, compared with the control, the outcome present group showed increased levels of HbA1C (7.3 ± 1.5 vs. 6.8 ± 1.4, *P* = 0.016), high rank of Gensini scores (45.1 ± 41.8 vs. 32.0 ± 35.2, *P* = 0.022) and mild high concentration of apoB (1.1 ± 0.3 vs. 1.0 ± 0.3, *P* = 0.048). Except the above three variables, the remained baseline characteristics between the two groups were roughly matched (All *P* > 0.05).

**Table 1:** Baseline characteristics of the study population

Variables	Outcome		*P*-value
	Present (*n* = 47)	Absent (*n* = 281)	
Risk factors			
Age (years)	59.2 ± 9.4	59.4 ± 9.3	0.891
Male gender	30 (63.8)	210 (74.7)	0.154
BMI (kg/m^2^)	25.9 ± 3.2	25.4 ± 3.0	0.067
Current smoking	21 (44.7)	160 (56.9)	0.153
Hypertension	36 (76.6)	226 (80.4)	0.557
Hyperlipidemia	95 (80.5)	77 (77.8)	0.557
PVD	0 (0.0)	6 (2.1)	0.599
Prior Stroke	1 (2.1)	12 (4.3)	0.702
Family history of CAD	3 (6.4)	32 (11.4)	0.444
LV-EF (%)	61.7 ± 9.5	62.1 ± 8.1	0.805
Laboratory test			
hs-CRP (mg/L)	6.8 ± 1.7	6.0 ± 1.4	0.805
Neutrophil Count (109/L)	3.9 ± 1.0	3.8 ± 1.2	0.797
Fibrinogen (g/L)	138.9 ± 16.6	137.2 ± 14.6	0.095
Hemoglobin A1C (g/L)	7.3 ± 1.5	6.8 ± 1.4	0.016
Creatinine (umol/L)	77.3 ± 15.9	76.2 ± 15.5	0.654
NT-pro-BNP (fmol/mL)	777.9 ± 736.0	731.8 ± 560.1	0.619
Lipid profile			
Remnant Cholestero (mmol/L)	0.6 ± 0.6	0.6 ± 0.4	0.390
Triglycerides (mmol/L)	1.9 ± 0.8	1.7 ± 0.9	0.072
Total Cholesterol (mmol/L)	4.2 ± 1.3	3.9 ± 0.9	0.170
LDL-C (mmol/L)	2.5 ± 1.0	2.3 ± 0.8	0.208
HDL-C (mmol/L)	1.1 ± 0.3	1.1 ± 0.3	0.981
Lipoprotein (a) (mg/L)	272.3 ± 286.1	240.1 ± 237.4	0.405
Apoprotein A-1 (g/L)	1.5 ± 0.3	1.4 ± 0.3	0.386
Apoprotein B (g/L)	1.1 ± 0.3	1.0 ± 0.3	0.048
Gensini scores (points)	45.1 ± 41.8	32.0 ± 35.2	0.022
DES Implantation	31 (73.8)	158 (68.7)	0.825
Medicine treatment			
Aspirin	46 (97.9)	274 (97.5)	1.000
Clopidogrel	45 (95.7)	269 (95.7)	1.000
Statin	47 (100.0)	272 (96.8)	0.368
Beta-blocker	42 (89.4)	229 (81.5)	0.218
ACE-I/ARB	30 (63.8)	213 (75.8)	0.105

*BMI* body mass index, *PVD* peripheral vascular disease, *CAD* coronary artery disease, *hs-CRP* high sensitivity C-reactive protein, *NT-pro-BNP* N-terminal pro-Brain natriuretic peptide, *LV-FE* left ventricular ejection fraction, *LDL-C* low density lipoprotein cholesterol, *HDL-C* high density lipoprotein cholesterol, *DES* drug eluting stent, *ACE-I* angiotensin converting enzyme inhibitors, *ARB* angiotensin receptor blocker

### Relation of remnant cholesterol to inflammatory markers and Gensini scores

**Fig. 1:**
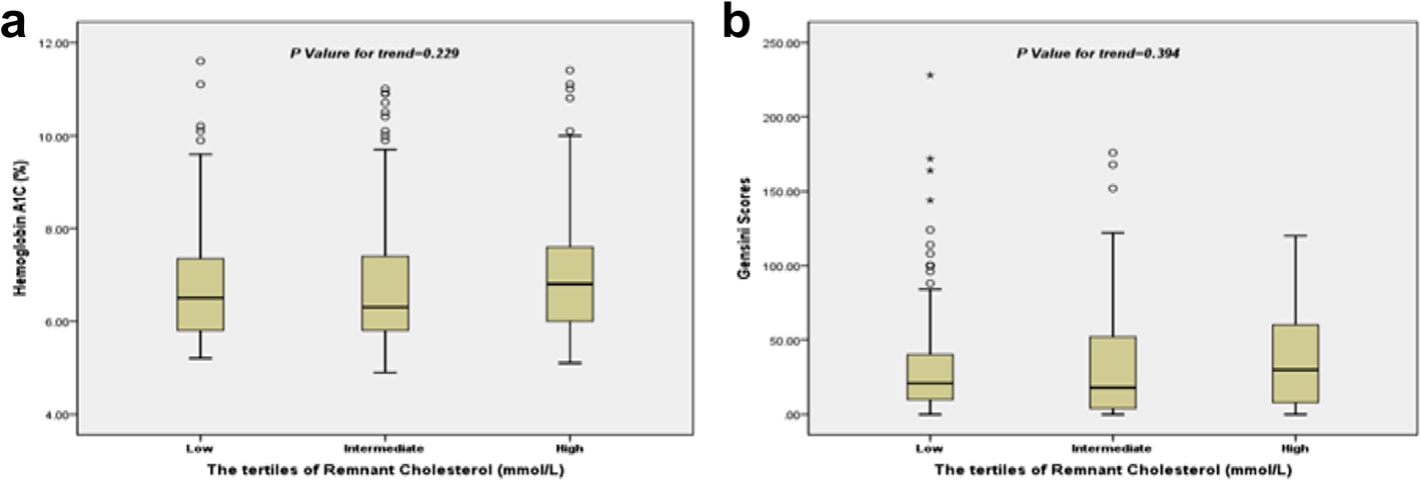
Correlation of remnant cholesterol with hemoglobin A1C and Gensini scores (**a** HbA1C; **b** Gensini scores)

According to Spearman’s correlation analysis, the baseline remnant cholesterol were positively associated with hs-CRP, neutrophils count, and fibrinogen (correlation coefficient of *R* ^2^ = 0.20, 0.12 and 0.14; *P* = 0.000, 0.036 and 0.010 respectively), but did not correlate with HbA1C and Gensini scores (All *P* > 0.05, Table 2). Similarly, the test for trend by Chi-Squared for levels of HbA1C and Gensini scores among the tertiles of remnant cholesterol was not significant (Fig. 1a and b).

**Table 2:** Spearman’s correlation of remnant cholesterol to inflammatory biomarkers and Gensini scores

Variables	Remnant cholesterol	Triglyceride	LDL-C	Lipoprotein (a)
hs-CRP	0.200; <0.000	0.069; <0.211	0.067; <0.224	0.068; <0.221
Neutrophil count	0.116; <0.036	0.105; <0.057	0.017; <0.761	0.093; <0.093
Fibrinogen	0.142; <0.010	0.023; <0.677	−0.022; <0.694	0.208; <0.000
HbA1C	0.081 ;<0.145	0.106; <0.055	0.040; <0.469	−0.033; <0.553
Gensini Scores	0.075; <0.178	0.115; <0.038	0.125; <0.023	0.035; <0.531

*hs-CRP* high sensitivity C-reactive protein, *HbA1c* glycosylated hemoglobin A1c, *LDL-C* low density lipoprotein cholesterol

Although remnant cholesterol was noticeably correlated to serum triglyceride (*R* ^2^ = 0.832, *P* = 0.000), they did not show same relation to the Gensini scores (*R* ^2^ = 0075 vs. 0.115, *P* = 0.178 and 0.038 respectively). As expected, the levels of LDL-C were associated with Gensini scores (*R* ^2^ = 0.125, *P* = 0.023), and the lipoprotein (a) correlated to fibrinogen (*R* ^2^ = 0.208, *P* = 0.000).

### Utility of remnant cholesterol in predicting short-term cardiovascular prognosis

**Fig. 2:**
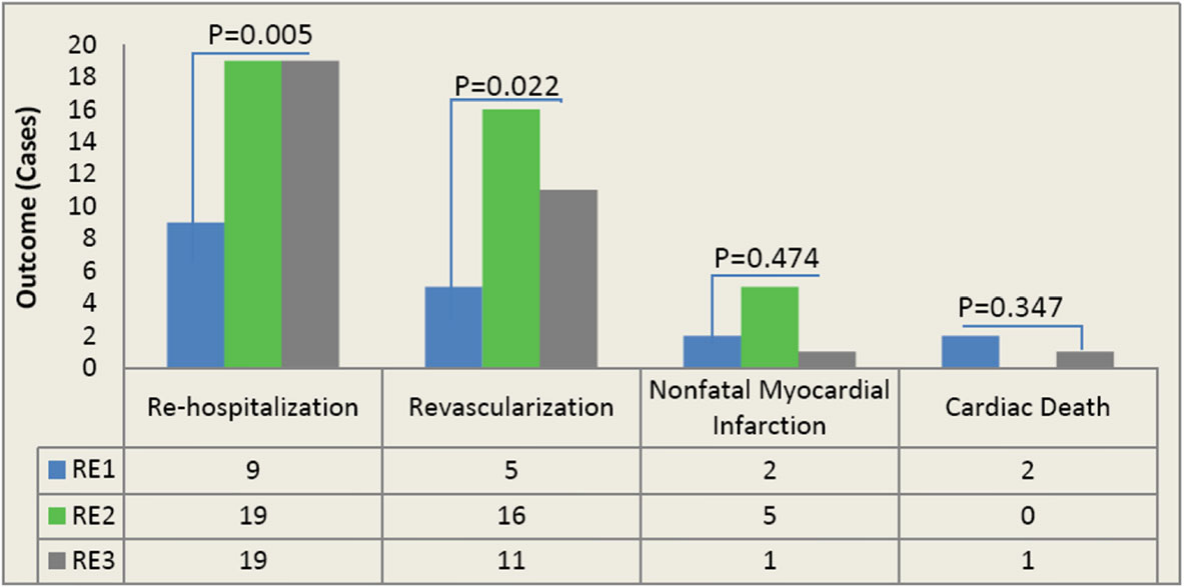
Correlation of remnant cholesterol with cardiovascular outcomes

During an average period of 12-month follow-up, 47 out of the 328 patients (14.3%) underwent adverse cardiovascular outcome (Fig. 2). The incident of endpoint events such as revascularization, re-hospitalization due to attack of angina pectoris and heart failure, none-fatal myocardial infarction, and cardiac death was 32 (66.7%), 5 (10.4%), 8 (16.7%), and 3 (6.2%) respectively.

**Fig. 3:**
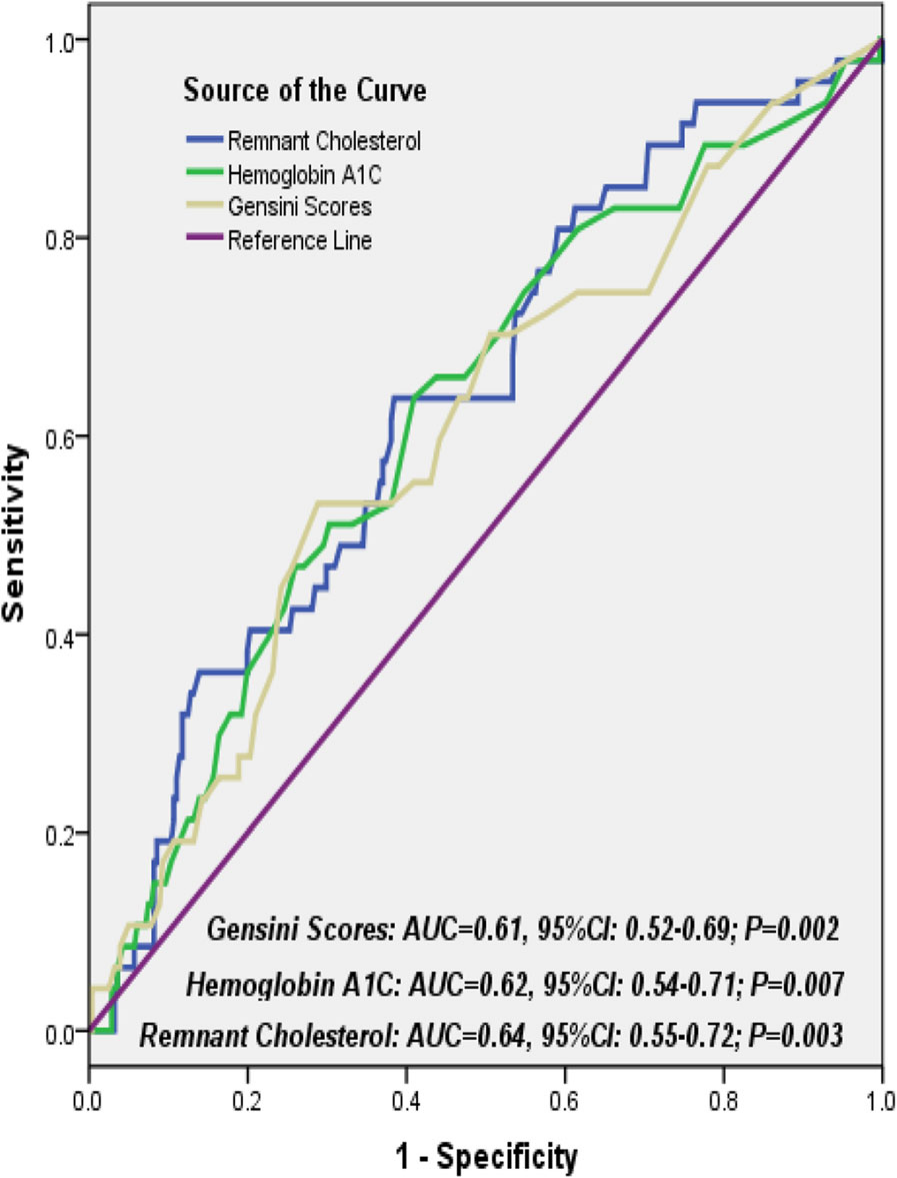
Receiver operating characteristic curves showed discriminatory power of remnant cholesterol, hemoglobin A1C and Gensini scores on cardiovascular outcomes

**Fig. 4:**
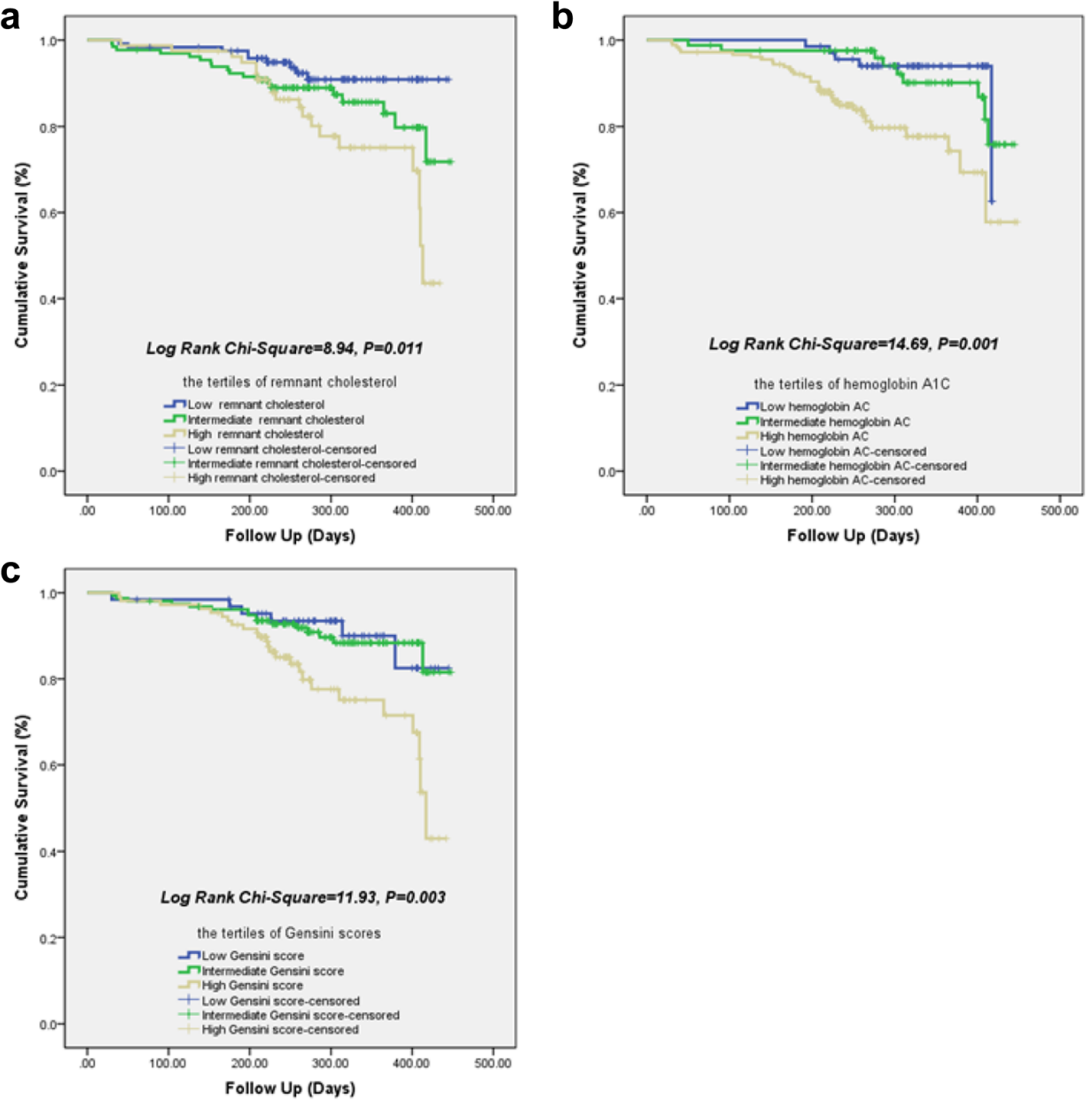
Kaplan-Meier curves for 12-month cumulative survival by the tertiles of remnant cholesterol (**a**), hemoglobin A1C (**b**) and Gensini scores (**c**)

As showed in Fig. 3, area under the ROC curves (AUC) indicated a strong discriminatory power of remnant cholesterol for the short-term cardiovascular outcomes (AUC = 0.64, 95% confidential interval [CI] =0.55–0.72; *P* = 0.003), with an optimal cutoff value of 0.34 mmo/L. Moreover, similar to HbA1C and Gensini scores, Kaplan-Meier curves for cumulative event-free survival based on the tertiles of admission remnant cholesterol suggested that the higher levels of remnant cholesterol showed relatively higher cardiac events for diabetics with new-onset stable CAD (Log rank Chi-Square = 8.94, *P* = 0.011) (Fig. 4a to c).

However, multivariate analysis of Cox proportional hazards regression (Table 3) indicated that, apart from HbA1C (Hazard ratio [H.R.] = 1.38, 95% CI: 1.14–1.66, *P* = 0.001) and Gensini scores (H.R. = 1.00, 95% CI: 1.00–1.02; *P* = 0.035), nonfasting remnant cholesterol did not qualify as an independent predictor of overall outcome in these settings (H.R. = 1.05, 95% CI: 0.46–2.37, *P* = 0.909).

**Table 3:** Cox proportional hazards regression analysis to determine the predictive value of remnant cholesterol for short-term cardiovascular outcome

Variables	Univariate		Multivariate	
	H.R. (95% CI)	*P*-value	H.R. (95% CI)	*P*-value
Gender	0.61 (0.33–1.10)	0.099	0.78 (0.36–1.66)	0.512
Age	0.99 (0.97–1.03)	0.941	0.98 (0.94–1.01)	0.195
BMI	1.06 (0.96–1.16)	0.266	1.06 (0.96–1.17)	0.239
Current Somking	1.68 (0.94–2.99)	0.080	1.48 (0.74–2.97)	0.273
Hypertension	0.68 (0.36–1.32)	0.255	0.79 (0.39–1.58)	0.507
Family history of CAD	1.61 (0.49–5.19)	0.427	1.77 (0.53–5.86)	0.352
Gensini Scores	1.01 (1.00–1.02)	0.004	1.00 (1.00–1.02)	0.035
Lipoprotein (a)	1.00 (0.99–1.00)	0.395	1.00 (0.99–1.00)	0.978
LDL-C	1.25 (0.90–1.74)	0.182	1.04 (0.73–1.47)	0.846
Remnant Cholesterol	1.38 (0.67–2.87)	0.378	1.05 (0.46–2.37)	0.909
Hs-CRP	1.02 (0.95–1.09)	0.548	0.98 (0.94–1.01)	0.407
Neutrophil Count	1.09 (0.86–1.40)	0.442	1.01 (0.77–1.32)	0.952
Fibrinogen	1.35 (1.01–1.79)	0.042	1.32 (0.84–2.07)	0.234
Hemoglobin A1C	1.37 (1.16–1.62)	0.000	1.38 (1.14–1.66)	0.001

*BMI* body mass index, *CAD* coronary artery disease, *LDL-C* low density lipoprotein cholesterol, *hs-CRP* high sensitivity C-reactive protein

## Discussion

To our knowledge, there have been no previous studies on determining whether the baseline levels of remnant cholesterol qualified for a useful predictor independent of traditional prognostic variables for adverse outcomes. The main findings of the present study were summarized as follows. Firstly, according to Speaman’s correlation, remnant cholesterol was positively associated with major inflammatory biomarkers such as high sensitivity C-reactive protein, neutrophil count and fibrinogen (*R* ^2^ = 0.20, 0.12 and 0.14; *P* = 0.000, 0.036 and 0.010, respectively). Secondly, although remnant cholesterol was significantly correlated to serum triglyceride (*R* ^2^ = 0.832, *P* = 0.000), they did not show approximate relation to the Gensini scores (*R* ^2^ = 0075 vs. 0.115, *P* = 0.178 and 0.038 respectively). Thirdly, the ROC curve indicated a matchable discriminatory power of remnant cholesterol, HbA1C and Gensini scores for the cardiovascular outcomes in the study population (AUC for remnant cholesterol, HbA1C and Gensini scores was 0.64, 0.62 and 0.61; *P* value was 0.003, 0.007 and 0.002). Moreover, Kaplan-Meier curves suggested that a higher concentrations of remnant cholesterol were more frequently accompanied with adverse cardiovascular events (Log rank Chi-Square = 8.94, *P* = 0.011). However, multivariate analysis in Cox regression model indicated that, apart from HbA1C and Gensini scores, the baseline remnant cholesterol did not qualify for an independent predictor of overall outcome in these settings (H.R. = 1.05, 95% CI: 0.46–2.37, *P* = 0.909). Obviously, the current study not only partially confirmed the hypotheses of previous studies, but also provided novel information concerning the role of non-fasting remnant cholesterol in predicting outcomes in diabetic patients with new-onset stable CAD.

Abundant evidence are indicating that the elevated levels of remnant cholesterol played an important role in the development of atherosclerosis, inflammatory and CAD [1, 5, 7, 23–25]. Patients cohort studies, in-vitro and animal investigations all support the conception that elevated levels of remnant cholesterol lead to atherosclerosis as the same way as elevated levels of LDL-C [26]. According to the serial studies from Varbo, et al., the hazards ratio for CAD in the highest quintile of remnant cholesterol (H.R. = 2.3, 95% CI: 1.7–3.1) or remnant cholesterol/HDL-C (H.R. = 2.6, 95% CI: 2.1–3.2) were greater than the lowest quintile population [7]. Furthermore, several genetic studies demonstrate evidence of remnant cholesterol’s being as a causative risk factor for CAD independent of HDL-C concentration [5, 23, 27–29]. Recent genetic studies with very large samples also indicate that elevated remnant cholesterol is associated with low-grade inflammation, whereas elevated LDL-C is not [1]. Coincided with previous studies, the baseline remnant cholesterol of current study are positively associated with major inflammatory markers such as hs-CRP, neutrophil count, and fibrinogen (*R* ^2^ = 0.20, 0.12 and 0.14; *P* = 0.000, 0.036 and 0.010 respectively). And as expected, the elevated of LDL-C is associated with Gensini scores (*R* ^2^ = 0.125, *P* = 0.023) but remnant cholesterol is not. However, during an average period of 12-month follow-up for the study population, 47 out of the 328 patients (14.3%) undergo adverse cardiovascular outcome and the revascularization are highlighted as the leading cause (66.7%). Therefore, we hypothesize that the elevated remnant cholesterol at admission may signify a more serious inflammatory state and atherogenic course. Besides, all the enrolled patients who have indication of revascularization received complete revascularization (mainly via percutaneous coronary intervention or implantation of drug eluting stent) during the first admission.

Furthermore, according to the ROC curve, non-fasting remnant cholesterol is suggested as a robust discriminator for the short-term cardiovascular outcomes of diabetics with new-onset stable CAD, with an absolute AUC mildly higher than AUC of HbA1C and Gensini scores (AUC of remnant cholesterol, HbA1C and Gensini scores is 0.64, 0.62 and 0.61 respectively; all *P* < 0.05). Meanwhile, similar to HbA1C and Gensini scores, Kaplan-Meier survivor curves suggest that the higher levels of remnant cholesterol show more incidence of cardiovascular events in these settings (Log rank Chi-Square = 8.94, *P* = 0.011). However, multivariate analysis in Cox regression model indicates that, apart from HbA1C and Gensini scores, the independent predictive value of baseline remnant cholesterol for cardiovascular events is attenuated after correction for associated variables and clinical characteristics (H.R. = 1.05, 95% CI: 0.46–2.37, *P* = 0.909). The underlying cause may consist at least of three aspects. Utmost, correlation of remnant cholesterol with low-grade systematic inflammation and CAD or events are not solely driven by diabetes mellitus or obesity [24]. Thus, the lack of causal association between remnant cholesterol and CAD or Gensini scores and events in diabetic participants can be explained by a lack of statistical significance. Moreover, prior basic study has suggested that genetic variants in diabetics are not enough to show causality in the presence of other risk factors [1]. Meanwhile, potent lipid-lowing therapy will attenuated the causal effect of remnant cholesterol and thus it may not lead to atherosclerosis in individuals of diabetics (100% patients of observation group in study treated with statin). Secondly, irrespective of fasting states, the circulating levels of remnant cholesterol have much greater variability than LDL-C [30]. Thirdly, compared with the control group, the observation group is accompanied with relatively higher HbA1C. Unquestionable, elevated levels of HbA1C implied not only severe disorder of glycolipid metabolism, but low-grade of systematic inflammatory response, vascular dysfunction and atherosclerotic progress in these settings as well [12, 31–34]. Nonetheless, although elevated remnant cholesterol have been increasingly prominent and gradually emerged as a leading impeller for cardiovascular disease and even LDL-C has been tailored to optimal levels, the powerful residual risks for diabetic population is far more than remnant cholesterol alone [5, 35, 36].

The limitations of our study are obvious. First of all, the sample size of the current study is relatively limited. Besides, the period of follow-up is comparably in a short duration and thus unavoidably leads to the bias for totally observing the outcome and/or the severity of CAD. Utmost, as a post hoc analysis, our investigation fail to set a strict control and direct compare the predictive power of remnant cholesterol for patients with and without diabetics. Finally, we do not determine the impact of genetic variants on remnant cholesterol and other lipids parameters, and subsequently on the overall outcomes.

## Conclusions

Non-fasting remnant cholesterol is associated with inflammatory biomarkers and incidents of coronary revascularization, but not qualified as an independent predictor for short-term prognosis of diabetics with new-onset stable CAD in the era of comprehensive revascularization and potent statin therapeutics. Further studies with larger sample assessing the predictive usefulness of the remnant cholesterol for the severity of CAD and its events in varied background need to be considered.
